# Facial Palsy in COVID-19 Patient: A Case Report

**DOI:** 10.31729/jnma.7890

**Published:** 2022-11-30

**Authors:** Shailes Paudel, Mijjal Gopal Shrestha, Rupesh Ramtel, Anayash Bhattarai, Umang Gupta

**Affiliations:** 1 Bhingri Primary Health Care Center, Bhingri, Pyuthan, Nepal; 2 Sumeru City Hospital, Dhapakhel, Lalitpur, Nepal; 3 Grande International Hospital, Dhapasi, Kathmandu, Nepal; 4 Maharajgunj Medical Campus, Maharajgunj, Kathmandu, Nepal

**Keywords:** *case reports*, *COVID-19*, *facial palsy*

## Abstract

Facial paralysis is one of the common problems leading to facial deformation. COVID-19 virus rarely has been shown to be associated with facial palsy. Here we present a case of a 60-year-old woman who presented with features of left lower motor facial palsy signs along with common features suggestive of COVID-19 infection. Brain imaging did not reveal any pertinent pathology but her polymerase chain reaction for COVID-19 was positive. This case highlights the fact that acute COVID-19 infection can be considered a cause of motor neuron facial palsy in the ongoing pandemic of COVID-19. Cases with neurological features suggestive of facial palsy therefore should be suspected of acute COVID-19 infection based on other pertinent findings of COVID-19 infection and thus polymerase chain reaction testing should be done.

## INTRODUCTION

Facial paralysis is a disfiguring disorder that has a considerable impact on the patient because it results in the loss of facial expression which is most commonly caused by a benign self-limiting inflammatory condition known as bell's palsy (BP).^1^ As new symptoms began to emerge in the Coronavirus disease (COVID-19) pandemic, neurological symptoms associated with this infection got the most attention. Along with other modalities to investigate facial nerve disorders, testing for COVID-19 infection should also be taken into consideration in selected patients. Peripheral facial palsy should therefore be added to the spectrum of neurological manifestations associated with COVID-19.^2^ We report a case of left lower motor facial palsy in a woman who tested positive for COVID-19 via Polymerase Chain Reaction (PCR) test.

## CASE REPORT

**Figure 1:**
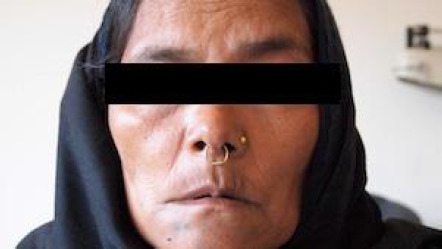
Deviation of the mouth towards the right side due to weakness on the left side of the face.

A 60-year-old female presented to the Emergency Department with complaints of facial deviation for the last 1 hour (Figure 1).

She also had a fever and non-productive cough for the last 4 days for which she was taking over-the-counter antipyretics and cough suppressants. She had no history of any chronic illnesses like hypertension, diabetes and tuberculosis. Examination of the case revealed the weakness of the left side of her face and forehead and thus deviating her face towards the right side. On initial presentation, the forehead was spared but later after about 6 hours of repeat examination, the forehead on the left side was also involved. Motor and sensory examination of upper and lower extremities were normal. Her vital signs were within normal limits.

A non-contrast Computed Tomography (CT) scan of the head was ordered and it did not reveal any underlying haemorrhage. Thus she was started on aspirin 75 mg once daily along with supportive care (paracetamol 500 mg thrice daily and antitussives). Repeat examination after 6 hours revealed no additional neurological findings and hence the diagnosis of bell's palsy was made.

Along with other routine investigations, her COVID-19 infection status was examined with PCR of her nasopharyngeal swab which came out to be positive. Chest X-ray did not reveal any lesion in her chest. She was then admitted to the isolation ward. Prednisolone 30 milligrams was added to her medication which was to be continued for 14 days and then planned for a tapered dose of prednisolone. Facial physiotherapy was advised which was to be continued for a week. Eye protective measures with artificial tear drop to be used frequently were also added.

On the third day of admission upon re-examination, there was an improvement in her weakness although no active facial movements were present. There was no fever for 24 hours, paracetamol was stopped and other supportive measures were continued.

She was reassessed on the 7th day of admission. Slight twitching of muscles was visualised while attempting facial movement. There were no signs of active infection. Repeat PCR for COVID-19 was done. It was negative this time. She was then prepared for discharge from the isolation ward. The steroid was continued along with eye protective measures and advised for physiotherapy of facial muscles.

## DISCUSSION

Facial paralysis can be central which results from the involvement of upper motor nerves above the pons or peripheral from involving lower motor nerves below the pons. Our case presented paralysis of the upper face suggestive of the peripheral type which is spared in the central type and is the key differentiating sign.^3^ Bell's palsy is an acute facial nerve palsy involving lower motor neuron (LMN) leading to weakness on one side of the face without any other neurologic abnormalities on examination.^3,4^ The aetiology of Bell's palsy is mostly unknown; however, viral infections such as Herpes Simplex Virus (HSV-1) and Herpes Zoster Virus (HZV) are seen to be associated with it.^4,5^ Recent studies have shown emerging cases of bell palsy in patients with COVID-19 infection. According to a study done out of 348088 COVID-19 infected patients, 153 patients had new onset of Bell's palsy. The present analysis found a higher incidence of BP in patients with COVID-19 (0.08%).^6^ This translates to approximately 82 per 100 000 patients with COVID-19. The rate of recurrent BP in patients with previous BP at the time of COVID-19 diagnosis was 8.6%.^6^ Patients with COVID-19 infection mainly present with fever, dry cough, dyspnea and myalgia but can also progress to respiratory distress requiring hospitalization.^7^ However, patients can also present with neurologic symptoms such as headache, dizziness, anosmia and dysgeusia.^8^ A study done among eight patients with COVID-19 developed peripheral facial palsy during infection. In three patients, facial palsy was the first symptom. Nerve damage resulted in mild dysfunction in five patients and moderate in three. SARS-Cov-2 was not detected in CSF by PCR in any of the samples. Seven out of eight patients were treated with steroids and all patients have complete or partial recovery of the symptoms.^2^

The pathogenesis of neurological involvement in COVID-19 patients is still not clear. Some of the proposed mechanisms include the affinity of viruses towards ACE-2 (Angiotensin Converting Enzyme) receptors. One of the structural proteins of Coronavirus is the spike protein (S protein) which is responsible for attachment, fusion and entry into host cells. S protein has a high affinity toward ACE-2 receptors. The expression of these ACE-2 receptors by glial cells and neurons makes the virus an entry site to the brain and its interaction also results in functional changes in ACE-2 receptors causing disruption of the steady state cytokine axis resulting in pro-inflammatory reaction, vascular endothelial injury and neural damage. COVID-19 virus can also directly invade the olfactory bulb and nerve resulting in central nervous system (CNS) entry and neural damage. Immune-mediated damages are also one of the mechanisms for neural injuries. During COVID-19 infection, there is a surge in proinflammatory cytokines resulting in a cytokine storm which damages the blood-brain barrier (BBB) and activates glial cells resulting in neuroinflammation and functional disruption.^5,9,10^ These mechanisms can impair and damage facial nerves resulting in bell's palsy.

The patient with bell's palsy presents with paralysis of one side of the face resulting in loss of facial crease and nasolabial fold, drooping of the corner of the mouth, spilling of food and saliva from the side of the mouth and tearing of eyes.^11^ In our case diagnosis was made clinically with detailed clinical history and neurological examination as the patient had a normal CT scan and did not give permission to perform magnetic resonance imaging (MRI), nerve conduction test and lumbar puncture.

Bell's palsy is a reversible condition and is managed with corticosteroids with or without antiviral medication. Administration of corticosteroid early within 3 days of the onset of symptoms have a positive outcome and speedy recovery.^3,5^ In our patient prednisolone 30 mg was started and was gradually tapered after which the patient's symptoms started to improve.

The above-presented case describes COVID-19 infection as one of the emerging causes of facial palsy. The treatment modality for this case has no significant difference as compared to conventional treatment of idiopathic facial palsy. The concurrent COVID-19 infection must be ruled out in patients presenting with neurological symptoms and other symptoms suggestive of COVID-19. Proper history taking with examination can be used as a guide to suspect COVID-19 in any patient.
